# Effect of deposit chemistry on microbial community structure and activity: Implications for under-deposit microbial corrosion

**DOI:** 10.3389/fmicb.2023.1089649

**Published:** 2023-02-09

**Authors:** Maria A. Diaz-Mateus, Silvia J. Salgar-Chaparro, Laura L. Machuca, Hanan Farhat

**Affiliations:** ^1^Curtin Corrosion Centre, WA School of Mines: Minerals, Energy, and Chemical Engineering, Curtin University, Bentley, WA, Australia; ^2^WA School of Mines: Minerals, Energy, and Chemical Engineering, Curtin University, Bentley, WA, Australia; ^3^Qatar Environment and Energy Research Institute (QEERI), Doha, Qatar

**Keywords:** multispecies consortium, sand-deposit, microbial community structure, functional profile, under-deposit microbial corrosion, carbon steel

## Abstract

**Introduction:**

The deposition of solid particles carried by production fluids from oil and gas companies in horizontal surfaces of different assets has shown to cause severe localised corrosion. Sand, one of the most common deposits in the energy sector pipelines, is frequently mixed with crude, oil, asphaltenes, corrosion inhibitors, and other organic compounds. For this reason, they might favour the metabolic activity of native microbial communities. This study aimed to determine the impact of sand-deposit chemical composition on the microbial community structure and functional attributes of a multispecies consortium recovered from an oilfield and the resulting risk of under-deposit microbial corrosion of carbon steel.

**Methods:**

Sand deposits recovered from an oil pipeline were used in their raw form and compared against the same deposits exposed to heat treatment to remove organic compounds. A four-week immersion test in a bioreactor filled with synthetic produced water and a two-centimeter layer of sand was set up to assess corrosion and microbial community changes.

**Results:**

The raw untreated deposit from the field containing hydrocarbons and treatment chemicals resulted in a more diverse microbial community than its treated counterpart. Moreover, biofilms developed in the raw sand deposit exhibited higher metabolic rates, with functional profile analysis indicating a predominance of genes associated with xenobiotics degradation. Uniform and localized corrosion were more severe in the raw sand deposit compared to the treated sand.

**Discussion:**

The complex chemical composition of the untreated sand might have represented an additional source of energy and nutrients to the microbial consortium, favoring the development of different microbial genera and species. The higher corrosion rate obtained under the untreated sand suggests that MIC occurred due to syntrophic relationships between sulphate reducers or thiosulphate reducers and fermenters identified in the consortium.

## 1. Introduction

Production fluids from oil and gas pipelines can carry different solid compounds including corrosion products, silt, sandstone, and solids from the formation reservoir (Brown and Moloney, 2017). During periods of low or intermittent flow, solid particles can sediment and form recalcitrant scales over the horizontal surfaces of the assets. Field observations have shown that the presence of deposits in the interior of the pipes or vessels can cause severe localized corrosion, a phenomenon known as under deposit corrosion (UDC) (Campbell, 2002; Durnie et al., 2005). Nevertheless, some laboratory studies have shown that the presence of deposits in a system can also hinder corrosion (Dugstad, 1998; Been et al., 2010; Echaniz et al., 2019; Grinon-Echaniz et al., 2021). These different outcomes have been attributed to the physical characteristics of the deposit (formation, composition, depth, age, etc.), mechanisms related to corrosion (diffusion, precipitation, microbial presence, etc.), and the associated water chemistry (water, oil, pH, salt content, etc.; Crolet, 1993; Zhu et al., 2018).

Currently, there is a scientific consensus that deposits formed in oil and gas equipment create suitable environments for bacterial colonization and biofilm development that can also cause microbiologically influenced corrosion (MIC) (Samant and Singh, 1998; Esan et al., 2001; Comanescu et al., 2016). This phenomenon has been called under deposit microbial corrosion (UDMC) (Machuca Suarez et al., 2019). To the best of the authors’ knowledge, there are only a few publications where UDMC was investigated in the presence of sand deposits. For instance, Liu et al. (2018) found that the sulfate reducing bacteria (SRB) *Desulfotomaculum nigrificans* enhanced the galvanic coupling between bare and deposit-covered steel, which resulted in acceleration of localized corrosion. Similarly, Suarez et al. (2019) demonstrated that a native thermophilic microbial consortium of methanogens, fermenting and sulphidogenic microorganisms increased eight times the average corrosion rates of carbon steel.

Sand is one of the most common deposits found in oil and gas pipelines. Sandstone reservoirs are among the main hydrocarbon sources worldwide, and influx can occur during perforation (Oyeneyin et al., 2005). Sand can also enter the pipelines during water injection (Wang and Melchers, 2017). For this reason, the effect of sand deposits on the severity of UDC has been extensively studied (Huang et al., 2010). However, it is noted that the common feature in these studies is the use of acid-clean commercial sand in the experiments, which does not represent deposits found in industrial environments. Oilfield sand deposits are commonly mixed with crude oil, resins, asphaltenes, waxes and corrosion inhibitors (Gieg et al., 2020), which contain heteroatoms of nitrogen (nitrates, nitrites), sulphur (thiosulphate, sulphate), carbon, and phosphorus that may serve as electron donors and nutrients for anaerobic microbial metabolism and influence the microbial activity of native communities (Harris et al., 2010).

Microbial ecology research in the oil and gas industry has shown that shifts in microbial community structure and metabolic capabilities occur in response to changes in the environment, such as nitrate injection (Vigneron et al., 2017), hydrocarbons presence (D’Ugo et al., 2021), usage of mixed corrosion inhibitors (Duncan et al., 2014). It is therefore plausible to assert that UDMC rates and mechanisms will differ when two chemically different types of sand-deposits are tested, mainly because of the impact that the sand-deposit chemistry would have in the microbial ecology dynamics.

This study was conducted to investigate the impact of sand deposit chemical composition on the taxonomic and functional attributes of a multispecies consortium. And, to assess its influence on the corrosion of carbon steel. Studying the risk of UDMC in presence of oilfield deposits, and the interactions of native microorganisms with them, is essential for improving the current corrosion management strategies used by the oil and gas industry. Through this type of studies, new risks that could have been overlooked in the past are identified, and, the importance of including microorganisms and field samples in corrosion laboratory-based experiments is highlighted.

## 2. Materials and methods

### 2.1. Oilfield sand sample

A total of 1 Kg of sand was collected from a high pressure (HP) separator at an Australian oil production facility experiencing sand accumulation inside the flow line. The system was under chemical treatment with a commercial imidazoline based corrosion inhibitor and a commercial chemical biocide containing Tetrakis (hydroxymethyl) phosphonium sulphate Benzyl-(C12-C16 Linear Alkyl) (THPS) and Dimethyl-Ammonium Chloride Formaldehyde (DACF). The samples were transported in a sterile anaerobic container under refrigerated conditions (4°C) to the laboratory for the recovery of native microorganisms and chemical characterization.

### 2.2. Microbial recovery and consortium preparation

Upon arrival, oilfield sand was immediately inoculated in different culture media to recover the oilfield microbial community to be used in corrosion studies. For this purpose, ten grams of sand were grown in 40 mL of four (4) selective culture media to target the growth of sulphide producing prokaryotes (SPP), acid-producing bacteria (APB), iron reducing bacteria (IRB), and methanogens (MET). Culture media were prepared following the standard method NACE TM0194 (2004); SPP media was prepared following the guidelines proposed elsewhere (Suarez et al., 2019). All culture media were sparged with a gas mixture of 20% CO_2_/80% N_2_ for 1 h to saturate the solution, and dispensed in serum vials capped with rubber stoppers crimped sealed and autoclaved. The inoculated tubes were incubated for 21 days at 40°C. The day of the experimental set up, an aliquot of each culture media that showed positive microbial growth was adjusted to a final concentration of 1.9 × 10^7^ cells/mL in a falcon tube. The falcon tube was centrifuged at 15,000 × *g* for 5 min to harvest cells from each culture media, finally, the four different pellets were combined in 5 mL of sterile PBS for the inoculation of the reactors. Molecular identification of the consortium was performed using 16S rRNA gene sequencing and the results were described elsewhere (Diaz-Mateus et al., 2021).

### 2.3. Sand treatment

Part of the oilfield sand was washed with ultrapure water (Milli-Q system, resistivity 18.2MΩcm) and roasted at 600°C for 3 h in a muffle furnace (Thermolyne Industrial Benchtop Muffle Furnaces, Thermo Fisher Scientific) to remove surface organic matter and used as treated sand (Tian et al., 2012). After the temperature treatment, the sand was cooled to room temperature inside the furnace; and, later stored under vacuum conditions for further characterization.

### 2.4. Sand characterization

#### 2.4.1. Fourier transform infrared spectroscopy

Both treated and untreated sand were analyzed using Fourier transform infrared spectroscopy (FTIR) to determine the functional groups present. For this, a diamond internal reflection element (Perkin-Elmer, Spectrum Two IR) was used. The wavelength range used for reading the spectra was 1,000 to 4,000 cm^−1^.

#### 2.4.2. Chemical characterization

Chemical analysis of treated and untreated sand (performed by a Eurofins, ARL) was carried out following US EPA, APHA (American Public Health Association) and in-house test methods. Analyzes included: Total petroleum hydrocarbons by Gas Chromatography-Flame Ionization Detector (GC-FID) (USEPA SW 846-8360B), Total organic carbon, by the high temperature combustion method (APHA 5310B), and total Nitrogen using an automated Colorimetric/Turbidimetric Aquakem System (APHA 4500).

### 2.5. Carbon steel coupons preparation

Carbon steel with elemental composition of (weight %): C (0.07–0.8), Mn (1.38–1.39), Si (0.16–0.68), S (0.01), *P* (0.01), Ni (0.01–0.03), Cr (0.09–0.23), Mo (0.03–0.06), Cu (0.06–0.11), V (0.02–0.06), Nb (<0.01), Ti (<0.01), Al (0.009–0.038), B (<0.0005), and Fe (balance) was cut into square coupons with a surface area of 5.29 cm^2^ including a weld in the center of the sample. Coupons were electro-coated with epoxy (Powercron 6000CX; PPG Industrial coatings) and only one surface of the samples was wet-ground to a 600-grit finish using *SiC* paper, to expose it to the tested sand. Subsequently, the samples were washed, rinsed with absolute ethanol, and dried under nitrogen gas. Coupons were finally sterilized by UV irradiation (15 min at each side).

### 2.6. Under deposit microbial corrosion test

Two different corrosion scenarios were assessed using 2-litre capacity glass cells. (1) UDMC with raw untreated sand, (2) UDMC with treated sand. Five coupon replicates were used to evaluate each scenario. Coupons were placed horizontally in custom-made glass containers (Φ40 × 20 mm) designed to ensure uniform deposition of a 20 mm layer of sand on the surface of the samples (Machuca et al., 2017). 55 ± 0.2 grams of sand (treated and untreated, accordingly) were deposited on top of the carbon steel coupons and tapped to achieve the same height in the five glass containers. The reactors were then connected to a filtered-sterilized gas line (20% CO_2_/80% N_2_ mixture) to maintain anaerobic conditions throughout the exposure period.

The test solution used was synthetic produced water with the following composition: NaCl 462 mM, CaCl_2_.2H_2_O 1.5 Mm, K_2_HPO_4_ 2.8 mM, NH_4_Cl 4.6 mM, KCl 4.6 mM, MgCl.H_2_O 2.4 Mm, D-glucose 5 mM, Na_2_SO_4_.5H_2_O 45 mM, Na_2_S_2_O_3_4.02 mM, Na-formate 14.7 mM, Na-lactate 5.8 mM, Na-acetate 4.9 mM, 1.4 g L − 1 bactocasamino acids (BD), Na-pyruvate 4.4 mM and, 1 l of ultrapure water (Milli-Q system, resistivity 18.2MΩcm). The solution pH was buffered with sodium bicarbonate and the initial pH was 7.3 ± 0.2 reflecting pipeline *in situ* conditions.

Microbial consortium cells (Section “Corrosion measurements and metal surface analysis”) were added to the reactors at a final concentration of 10^7^cells mL^−1^. The temperature and stirring of the reactors were set to 40°C ± 1°C, and 200 rpm, respectively. To maintain an active microbial consortium throughout the exposure, reactors were maintained under batch feeding (30% of test solution replenished every 4 days). The test exposure period was 4 weeks.

### 2.7. Analytical methods

#### 2.7.1. Corrosion measurements and surface analysis

Corrosion measurements were performed on triplicate coupons retrieved from each reactor to determine uniform and localized corrosion rates. For this, the metal samples were washed with Clarke’s solution, following the ASTM G1 (2003) standard guidelines. Various sonication cycles of 1 min were carried out to completely remove the corrosion products that were strongly adhered to the metal. Afterwards, the weight of the sample was measured and corrosion rates were estimated from weight loss (ASTM G1, 2003). To obtain a localized corrosion rate, the surface of the coupons was analyzed with a 3D optical profilometry (Alicona InfiniteFocus G4). The deepest point present in each metal surface was used to calculate the pitting rate as described in the NACE standard practice SP-0775 (NACE SP0775, 2013). Coupons were also visualized using a Neon Dual-Beam field emission scanning electron microscope (FESEM) at an emission voltage at 15 kV.

#### 2.7.2. Microbial community composition and structure

The microbial community that thrived in the sand deposits of the two reactors was identified by 16S rRNA gene sequencing. Three replicates were characterized from each reactor. For this, at the end of the UDC test, the sand layer covering the carbon steel coupons was immersed in flasks containing 20 mL of anaerobic PBS and sonicated for 10 s followed by 15 s on ice, repeating for 5 cycles to detach sessile microorganisms from the sand grains. After recovering a total volume of 100 mL of PBS containing detached cells, 90 mL of the solution were centrifuged at 15,000 × *g* for 15 min to concentrate the pellet. Pellet was used for DNA extraction using a FastDNA™ SPIN Kit for Soil (MP Biomedicals) following the manufacture’s procedures. DNA concentration was verified using a Nanodrop spectrophotometer (NanoDrop™ Lite Spectrophotometer). The V3-V4 hypervariable region of 16S rRNA genes in the extracted DNA was amplified by PCR using the universal primers 341F (5’ CCTAYGGGRBGCASCAG3’) and 806R (5’ GGACTACNNGGGTATCTAAT 3′; Salgar-Chaparro et al., 2020a).

PCR products were sent to the Australian Genome Research Facility (AGRF) for library preparation and sequencing. PCR amplicons were generated using the primers 341F (50’ CCTAYGGGRBGCASCAG 3′) and 806R (5’GGACTACNNGGGTATCTAAT 3′; Yu et al., 2005). Thermocycling was completed with an Applied Biosystem 384 Veriti and using AmpliTaq Gold 360 master mix (Life Technologies, Australia) for the primary PCR. A secondary PCR to index the amplicons was performed with TaKaRa Taq DNA Polymerase (Clontech). The resulting amplicons were cleaned again using magnetic beads, quantified by fluorometry (Promega Quantifluor) and normalized. The equimolar pool was cleaned a final time using magnetic beads to concentrate the pool and then measured using a High-Sensitivity D1000 Tape on an Agilent 2,200 TapeStation. The pool was diluted to 5 nM and molarity was confirmed again using a High-Sensitivity D1000 Tape. This was followed by sequencing on an Illumina MiSeq instrument with a V3 (600cycles) kit (Illumina).

The Quantitative insights Into Microbial Ecology Software (Qiime2-DADA2 v. 2020.8.0 pipeline) was used for the analysis of the raw data (Pilloni et al., 2022; Rajala et al., 2022). The “dada2 denoise-paired” plugin was implemented for quality filtering, denoising and chimera removal of the amplicon sequences (Callahan et al., 2016). Parameters “—p-trim-left-f 10” and “—p-trim-left-r 10” were used to trim off the first 10 bases of both forward and reverse reads. Parameter “—p-trunc-len-f 280” was used to truncate the forward sequences at position 280. Parameter “—p-trunc-len-r 220” was used to truncate the reverse sequences at position 220, based on the demux-summary.qzv file (Salgar-Chaparro et al., 2020c). Filtered sequences were classified using BLAST (“feature-classifier classify-blast”) against the SILVA database version 138 and clustering at 90%. The taxonomic composition of each sample was illustrated using OriginPro.

#### 2.7.3. Microbial community alpha diversity analysis and functional capability

Estimates of bacterial community richness, diversity, and evenness were performed in Qiime2 v. 2020.8.0, using the “core-metrics-phylogenetic” method for obtaining the Chao1, Shannon and Simpsons diversity indices (Prodan et al., 2020). To account for differences in sequencing effort, all samples were rarefied to the lowest number of reads obtained from an individual sample (12388) prior to analysis.

The functional profile of the two different microbial communities in the two sand deposits was predicted from the obtained 16S rRNA gene data, using the R-based tool Tax4fun2 R, and based on KEGG level 2 category (Coclet et al., 2021). The results in percentages represent the fraction of the microbial community that possesses each specific functional capability. Linear discriminant analysis (LDA) effect size (LEfSe) (Segata et al., 2011) was performed to reveal the specific metabolic pathways significantly associated with treated and untreated sand. A size effect threshold of 4.0 on the logarithmic LDA score was set for discriminative metabolic pathways as significant biomarkers. A value of *p* of ≤0.05 was considered significant for statistical methods. LefSe analysis was performed online in the Galaxy workflow framework (Goecks et al., 2010).

#### 2.7.4. Microbial activity

The remaining 10 mL of cell suspension (Section “Microbial community composition and structure”) were used to evaluate metabolic activity levels of the sessile bacteria community in the two conditions. The concentration of the adenosine triphosphate (ATP) molecule was measured by luminescence after reaction with luciferase using the Quench-GoneOrganic Modified (QGO–M) test kit (Luminultra Technologies Ltd.), following the manufacturer’s instructions. Three different samples from each reactor were used for this analysis. ATP measurements were collected using the PhotonMaster™ Luminometer (Luminultra Technologies Ltd.), and ATP concentration (ng/g) was calculated from the measured luminescence by comparing it against a standard.

### 2.8. Statistical analysis

Statistical analysis of corrosion and adenosine triphosphate (ATP) data were conducted using SPSS 27 and PAST (V4.10). The statistical analyzes applied were selected based on the normality of the data in each variable. Shapiro–Wilk method was used to test the normality of the data in each variable. Then, one-way analysis of variance (ANOVA) with Tukey’s post-hoc means separation test was implemented to test the homogeneity of variances in each variable and identify statistically significant differences between variables normally distributed. Statistical comparison of microbial alpha diversity levels between the two sand deposits was calculated on rarefied data with a parametric *t*-test. Results of statistical tests were considered significantly different with value of *p* ≤0.05.

## 3. Results

### 3.1. Sand characterization

#### 3.1.1. Fourier transform infrared spectroscopy

**Figure 1:**
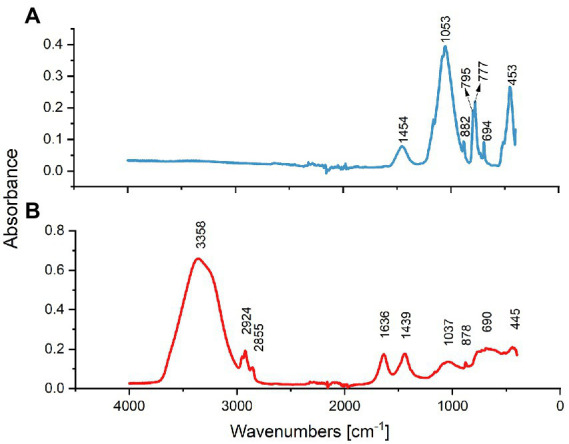
FTIR spectral analysis of treated and untreated sand samples. **(A)** Spectrum of treated sand indicating that SiO_2_ formed the sample predominantly. **(B)** Spectrum of the untreated oilfield sand indicating the presence of O-H and C-H functional groups.

FTIR results of treated and untreated sand are shown in Figure 1. Results showed common bands assigned to silicon dioxide and some silicates in both samples. The broad peaks seen at 1037 and 1,053 cm^−1^ in untreated and treated sand, respectively, consists of the Si-O-Si bond (Oh 2010). The Si–O symmetrical stretching vibrations observed at 795 and 777 cm^−1^ in the treated sand, the Si–O asymmetrical bending vibration at 445 ~ 453 cm^−1^, and the symmetrical bending of the Si–O group at 690 ~ 694 cm^−1^, indicates that the silica was in the form crystalline quartz in both samples (Anbalagan et al., 2010).

Although the main chemical matrix of both sand samples was similar, the FTIR spectra analysis showed key differences in some functional groups present in the untreated sand and absent in the treated sand. The peaks seen in the untreated sand sample (Figure 1B) at 2,924 and 2,852 cm^−1^ are indicative of the antisymmetric and symmetric stretching C-H bond, respectively, characteristic of the functional group of alkanes (Durnie et al., 2001). The FTIR spectral bands in the region 1,636 cm^−1^ suggest the presence of the C=C stretching vibration in alkenes in untreated sand (Gao et al., 2011; Patty et al., 2017). Moreover, the wide band in the range of 3,700 to 3,000 cm^−1^ (includes the characteristics bands at 3,678, 3,410, 2,928, and 2,853 cm^−1^) is attributed to the –OH stretching vibration that corresponds to the sinalol functional groups (Si-OH) and also to the physical absorbed water by the sand deposit (Sun et al., 2018).

The FTIR spectra indicated that the roasting of oilfield sand, removed the organic compounds present in the sample and caused a dihydroxylation of the sand.

#### 3.1.2. Chemical characterization

The chemical characterization of the treated and untreated sand samples is shown in Table 1. A clear difference in the chemistry of the two different sand samples was evidenced. Untreated sand contained higher levels of total petroleum hydrocarbons (TPH), organic carbon (TOC), total nitrogen and total Kjeldahl nitrogen. Findings of hydrocarbons by chemical characterization in the untreated sand corroborated the results of FTIR.

**Table 1:** Chemical composition of treated and untreated sand used as deposits in the UDMC test.

Compound	LOR^a^	Sand	
		Untreated	Treated
TPH C6-9 (mg/kg)	0.2	1.7	<0.2
TPH C10-14 (mg/kg)	0.2	490	<0.2
TPH C15-28 (mg/kg)	0.4	2,300	<0.4
TPH C29-36 (mg/kg)	0.4	630	<0.4
TPH C > 36 (mg/kg)	0.4	190	<0.4
Sum of TPH (mg/kg)	1.6	3,600	<1.6
TOC (%)	0.1	0.59	0.14
Total Kjeldahl Nitrogen (mg/kg)	10	56	43
Total Nitrogen (mg/kg)	10	56	46

^a^LOR, Limit of detection.

### 3.2. Corrosion measurements and metal surface analysis

#### 3.2.1. General corrosion

**Figure 2:**
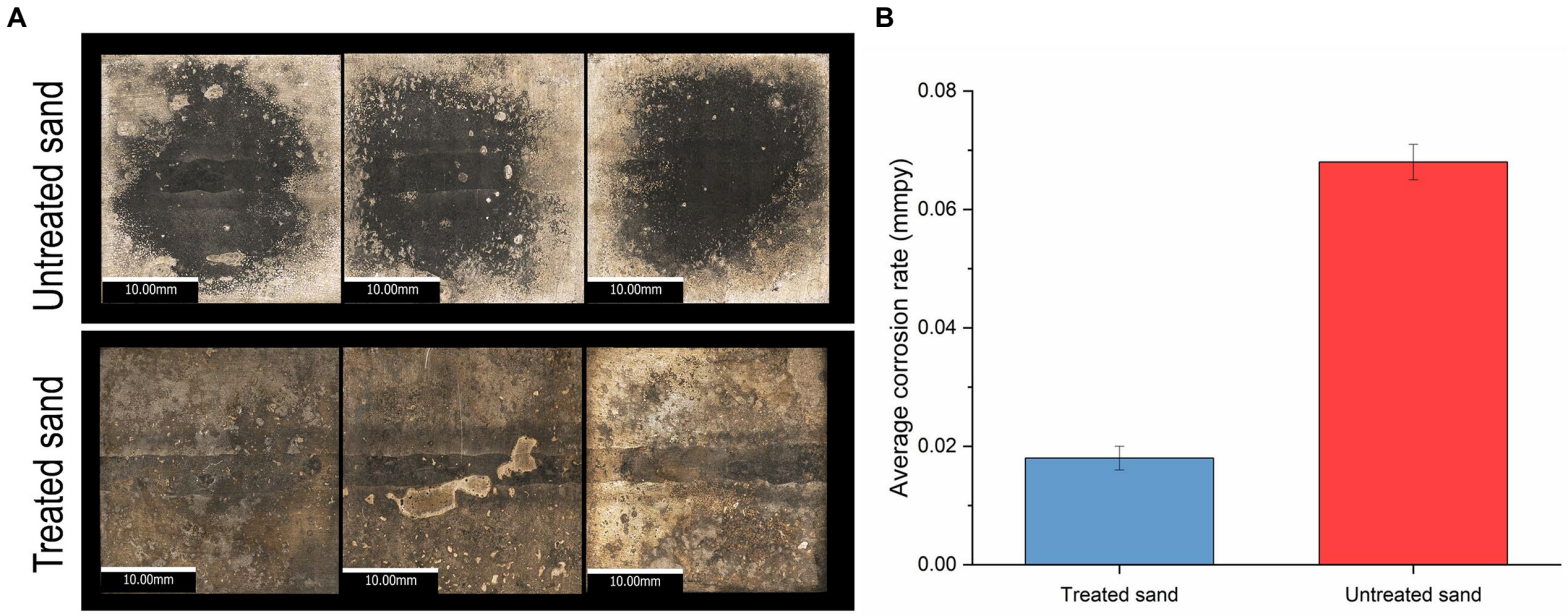
Uniform corrosion of carbon steel in a 4 weeks UDMC test with two chemically different sand deposits. **(A)** Visible-light microscopy images of metal samples at 5X resolution. **(B)** Average general corrosion rates by weight loss.

Metal surface imaging showed that the carbon steel exposed to untreated sand (Figure 2A) suffered severe localized corrosion in the form of a large cavity in the center of the sample, covering almost all the welded area, whereas the carbon steel samples exposed to treated sand showed mainly uniform corrosion. Corrosion rates by weight loss presented in Figure 2B showed that microorganisms developed within the treated sand deposits led to lower general corrosion rates of carbon steel in comparison with the corrosion rates obtained when microorganisms were interacting with the untreated sand deposit. Differences between corrosion rates of 0.068 mmpy in untreated sand versus corrosion rates of 0.018 mmpy in treated sand were statistically significant (*p* ≤ 0.05, Supplementary Table S1).

#### 3.2.2. Localized corrosion

**Figure 3:**
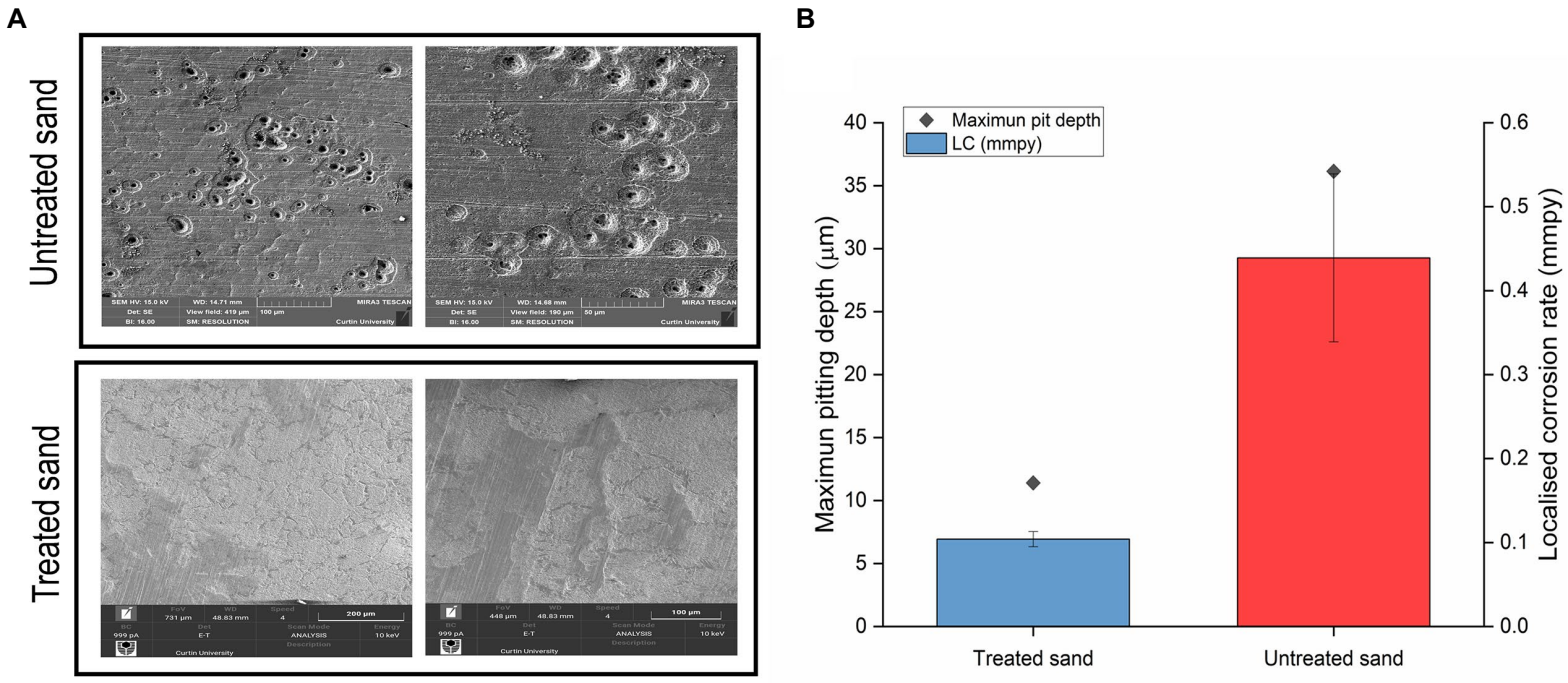
Localized corrosion analysis of carbon steel in a 4 weeks UDMC test with two chemically different sand deposits. **(A)** Scanning electron micrographs of steel surfaces after cleaning. **(B)** Localized corrosion rates calculated from the maximum pit depths.

After removing the sand, corrosion products and biofilm, the morphology of carbon steel samples surface was analyzed by SEM, results are shown in Figure 3A. They key difference observed was that coupons exposed to treated sand showed signs of general corrosion, whereas the coupons exposed to untreated sand suffered mainly localized corrosion in the form of deep pits.

3D optical profilometry was performed on three cleaned coupons for each test condition to assess the metal penetration in each treatment (Supplementary Figure S1). Maximum pitting depth and pitting rates are shown in Figure 3B. Pitting rates of 0.43 mmpy were found in carbon steel samples exposed to the untreated sand deposit whereas pitting rates of 0.10 mmpy were found in carbon steel exposed to the treated sand. Differences in the pitting rates between the two scenarios were statistically significant (*p* ≤ 0.05, Supplementary Table S2). According to the qualitative categorization of carbon steel corrosion rates established in the NACE standard practice NACE SP0775 (2013), the pitting rates in untreated sand are classified as severe, whereas pitting rates in treated sand are classified as low.

### 3.3. Microbiological analysis of sessile community in both treated and untreated sand

#### 3.3.1. Microbial composition, richness, diversity, and evenness

**Figure 4:**
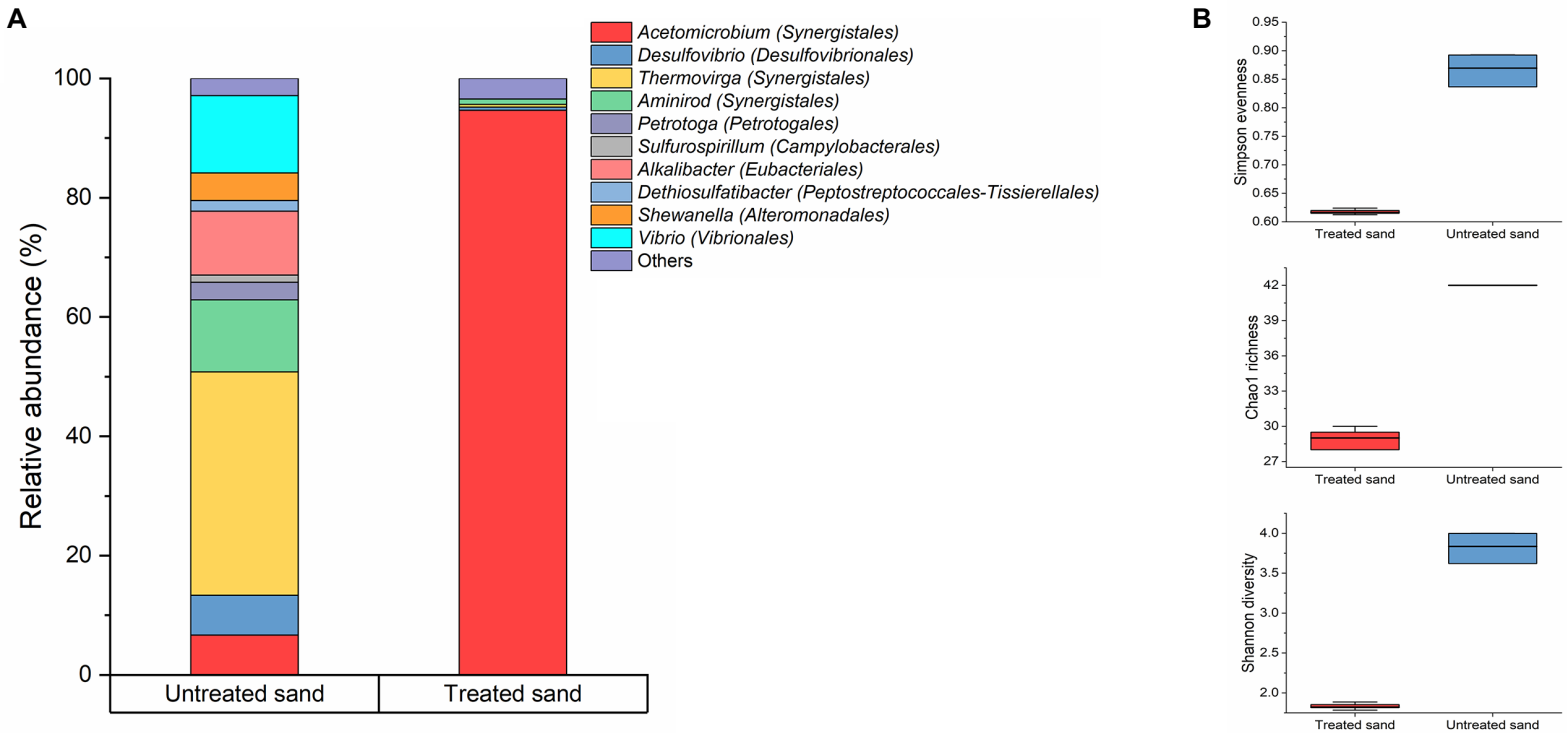
Microbial community changes driven by the sand-deposit chemical composition. **(A)** Community structure. Results show the mean relative abundances of microbial community classified at the genus level (*n* = 3). Phylogenetic order is indicated in parentheses. Bacterial genera with relative abundances >1% are shown; genus contributing ≤1% were presented as “others.” **(B)** Alpha diversity analysis. Boxes represent the interquartile range (IQR) between the first and third quartiles (25th and 75th percentiles, respectively), and the horizontal line inside the box defines the median. Whiskers represent the lowest and highest values.

The microbial community composition at genus level of the treated and untreated sand deposits is shown in Figure 4A. The microbial composition analysis revealed that the microbial populations established at each deposit were markedly different. In the treated sand, where organic compounds were removed, fermenting species from the genera *Acetomicrobium* were found as the predominant microorganisms in the population with a percentage of abundance of 95.7%, accompanied by sulphate reducers (*Desulfovibrio*), thiosulphate reducers (*Shewanella*), iron-oxidizers (*Pseudomonas*), and other fermenters (*Thermovirga* and *Caminicella*), with relative abundances less than 1% in the three samples analyzed. Contrarily, in the untreated field sand, fermenting microorganisms from the genera *Thermovirga* (37%), *Vibrio* (13%), *Aminirod* (12%), and *Alkalibacter* (10.6%), and *Acetomicrobium* (6.6%) accounted for the 79% of the community. In addition, thiosulfate reducers (*Shewanella*, *Dethiosulfatibacter*, *Petrotoga*), sulfate reducers (*Desulfovibrio*), and, nitrate reducers (*Sulfurospirillum*), were found with abundances higher than 5%. The other 17 microbial genera found in lower abundances (less than 1%), accounted for the 2.9% of the total microbial community. Differences found based on the sand deposit chemical composition reflect the dynamic interaction among the microorganisms that make up a community in response to the close surface (environment) they are interacting with. It is important to clarify that in order to handle uncertainties in the experiment, coming from the untreated sand, the samples were characterized using 16S rRNA gene sequencing. Results indicated that there were two predominant genera (*Thermovirga* and *Caminicella*) in the community. These predominant genera were also identified in the pooled microbial consortium inoculated to the bioreactors.

The comparison of the alpha diversity of the sessile communities established in the two chemically different sand deposits is showed in Figure 4B, and statistically significant differences in the gross community structure were found (*p* ≤ 0.05, *t*-test, Supplementary Table S3). The richness index (Chao1), and diversity index (Shannon) which consider the number of species in the community, were higher for the microbial community developed in the untreated sand and lower for the microbial community developed in the treated sand deposit. Likewise, the Simpson evenness, which represents the probability that two randomly selected individuals will be of the same species, showed that the community hosted in the untreated sand were more evenly distributed (between 0.83 and 0.89) than the one found in the treated sand (between 0.61 and 0.62; Figure 4B).

#### 3.3.2. Predicted functional profile

**Figure 5:**
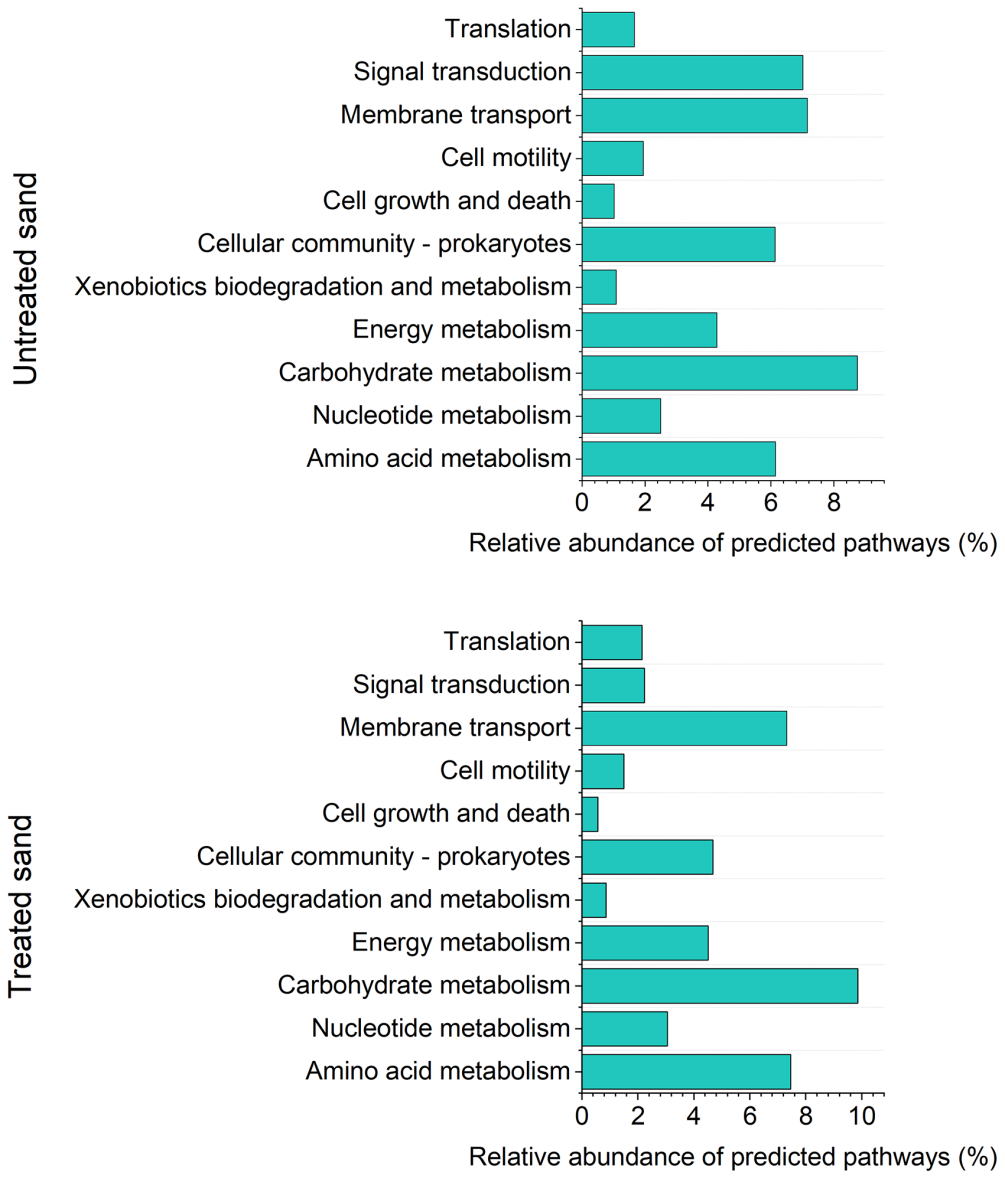
Analysis of predicted KEGG metabolic pathways at level 2, based on Tax4fun2: **(A)** Relative abundance of predicted pathways in sessile community from untreated sand. **(B)** Relative abundance of predicted pathways in sessile community from treated sand.

A total of 307 KEGGs (functional orthologs) were predicted across both samples, and used for the functional analysis at level 2 (Figure 5). A higher abundance of genes involved in amino acid metabolism, carbohydrate metabolism and membrane transport were predicted in the microbial community grown in the treated sand deposit. Contrarily, the relative abundance of genes involved in cell growth and death, signal transduction (transmission of molecular signals from the microorganism exterior to its interior to ensure an appropriate response), xenobiotics degradation and metabolism were predicted in lower abundance compared with the microbial community grown in the untreated sand. LefSe analysis indicated that 134 of the 307 pathways found, were significantly different between communities developed in the two chemically different sand deposits (Supplementary Table S4). LefSe analysis at level 3 showed that metabolic pathways related to the two-component regulatory system (proteins of adaptation to the environment for survival), biofilm formation, sulphur and nitrogen metabolism, and xenobiotics degradation were biomarkers of the community developed in the untreated sand. Whereas carbohydrate and amino acids metabolism, and, quorum sensing were biomarkers of the microbial community developed in the treated sand.

#### 3.3.3. Microbial activity

**Figure 6:**
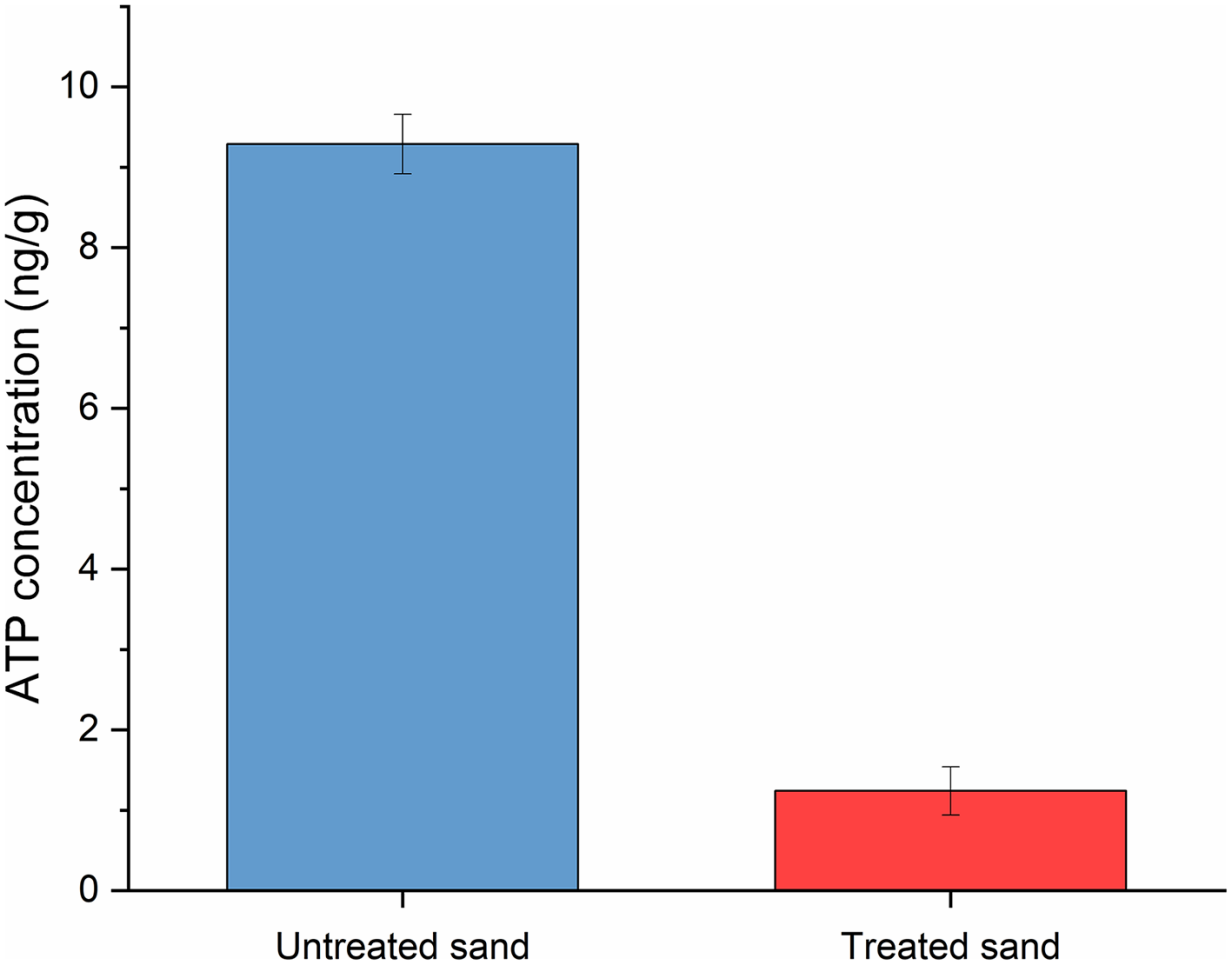
Adenosine triphosphate (ATP) concentration of biofilms growth within two different sand deposits in the UDC reactors after 4 weeks of immersion.

The concentration of cellular ATP of the sessile microorganisms developed within the sand deposits after UDMC tests is shown in Figure 6, results are presented as the mean ± standard deviation. One-way ANOVA analysis of the concentration of adenosine triphosphate in sessile microorganisms (by triplicate) confirmed that the microbial community developed within the 20 mm layer of untreated sand (9.29 ng/g) was significantly more active than the microbial community developed in the treated sand (1.24 ng/g; *p* ≤ 0.05; Supplementary Table S5).

## 4. Discussion

### 4.1. Effect of sand deposits chemistry on microbial community structure, activity levels, and functional profile

The sessile microbial community developed in both treated and untreated sand was dominated by fermenting microorganisms, despite the initial inoculum was heavily dominated by sulfate reducing, thiosulfate reducing and iron oxidizing bacteria, with lower abundance of fermenters. These results suggest that the fermenters present in the consortium had a stronger competitive preference for the carbon sources available in the test solution. Other authors have described similar results where the influence of carbon sources in the microbial community structure of a native sediment consortium containing iron-reducing bacteria, sulfate reducing bacteria and fermenting organisms was studied. Authors found that the microbial community shifted to a community dominated by fermenting organisms in cultures enriched with glucose and lactate as carbon sources (Lentini et al., 2012).

Significant differences were seen in the relative abundances of the predominant genera in the sessile communities developed at each condition. One of the main differences found is the increased abundance of *Acetomicrobium* in the treated sand, which resulted in almost the total make-up of the community. Although little is known about the genus *Acetomicrobium*, the few reported species belonging to this have shown higher growth rates when glucose is present in the culture medium (Soutschek et al., 1984). Though, this obligate anaerobe has wide metabolic plasticity and can ferment other substrates such as amino acids, dicarboxylic acids, and, other sugars (maltose, fructose), besides, it can use several sulphur compounds as terminal electron acceptors (Cook et al., 2018). These broad metabolic capabilities may have influenced in the establishment of this genera as the main group in the final microbial community in the treated sand deposit.

Aside from *Acetomicrobium* wide metabolic plasticity, the presence of residual biocide in the untreated sand might have also played a role in the significant differences evidenced in the microbial community developed in the sand samples. Although the organic compounds detected in the FITR spectra (Figure 2) cannot be identified with this technique, we know that biocide injection was an ongoing MIC mitigation strategy in the oilfield from where the sand sample was taken. Thus, it is possible that the dominant presence of *Acetomicrobium* in the reactor where sand depleted from organic compounds is due to the growth inhibition effect that the biocide was having on that population in the oilfield from where sand samples were obtained; and ceased when the biocide was removed from the system. Nonetheless, further analysis will be required to confirm susceptibility of *Acetomicrobium* to the field biocides.

Conversely, in the untreated sand, fermenters were mainly represented by five different genus (*Thermovirga*, *Vibrio*, *Aminirod, Alkalibacter*, and *Acetomicrobium*), coexisting with sulfate and thiosulfate reducers. Syntrophic interactions between fermenters and SRB have been reported in different anaerobic environments, as fermenters’ metabolic by-product H_2_ can act as an electron donor for the sulfate and thiosulfate reduction by SRB and thiosulfate reducing bacteria (TRB) (Dar et al., 2008). Considering that the only difference between the untreated and treated sand was its chemical composition (as the same carbon sources and electron acceptors were supplied in the test solution), it is inferred that the heteroatoms of nitrogen, sulphur, carbon, oxygen and phosphorous present on the hydrocarbons, residual biocide and corrosion inhibitors, represented an additional source of energy and nutrients to the microbial consortium inoculated in the reactor. This enriched environments, then led to a more diverse community, which was supported by the higher metabolic rates (ATP concentration) found in the untreated sand.

Alpha diversity analysis of the microbial communities established in the treated and untreated sand at the end of the 4 weeks of immersion confirmed that differences in the two communities biodiversity were significant, and that the untreated oilfield sand allowed the development of diverse microbial communities that resembled more the microbial communities usually found in oil and gas facilities and reservoirs (Machuca Suarez and Salgar-Chaparro, 2018; Suarez et al., 2019; Nicoletti et al., 2022). It has been reported that the predominant genera in the untreated sand, *Thermovirga*, possess homologs of the benzyl succinate synthase gene (*bssA*), which codes for a benzyl succinate synthase, the key enzyme of anaerobic toluene degradation, indicating that members of this genera may play a key role as primary fermenter in the anaerobic degradation of hydrocarbons (Vigneron et al., 2017). Similarly, *Aminirod* (also present in higher abundance in the community) has shown the ability to act as a secondary fermenter in the degradation of hydrocarbons. This strain can ferment propionate and butyrate (metabolic by-products generated by primarily fermenters) into acetate and H_2_ (Liu et al., 2021). Hence, our results support that the hydrocarbons present in the untreated sand (Table 1) were potentially being degraded by *Thermovirga* and *Aminirod* in a syntrophic metabolism. In contrast, genes related with carbohydrate, aminoacids and pyruvate metabolism were found as biomarkers in the microbial community developed in the treated sand.

### 4.2. Effect of sand deposits chemistry on under deposit microbial corrosion

Results from this study demonstrated that the chemical differences of the sand deposits tested lead to different under deposit microbial corrosion rates. Despite the differences in the relative abundances of the genera found in both treated and untreated sand, fermenting organisms, previously related to MIC were the predominant bacteria of the consortium (de Paula et al., 2014; Salgar-Chaparro et al., 2020b; Cai et al., 2021). *Acetomicrobium*, the dominant genus found in the treated sand microbial community (94.3%) uses the fermentation of simple sugars and amino acids as primary metabolic strategy, releasing corrosive metabolites such as acetic acid, CO_2_ and H_2_ to the environment; moreover, it possess the ability to reduce thiosulfate, elemental sulphur and cysteine to hydrogen sulphide (Maune and Tanner, 2012; Cook et al., 2018), which acidifies the water, causing pitting corrosion to carbon steel pipelines. Similarly, *Thermovirga*, the most abundant genus in the untreated sand microbial community (94.3%) is a thermophilic bacterium capable of fermenting proteins, organic acids, and single amino acids, producing ethanol, H_2_, and CO_2_ as metabolic by-products. *Thermovirga*, just as *Acetomicrobium* can also couple fermentation with the reduction of elemental sulphur (S_0_) to hydrogen sulphide (H_2_S) (Dahle and Birkeland, 2006; Duncan et al., 2009). It has to be noted that despite sulphide concentration was not measured during the test, a characteristic smell of H_2_S and the presence of a black cover in the sand deposits at the end of both test (suggesting FeS formation) suggests the formation of H_2_S in the tests.

The statistically significant differences in the corrosion damage generated by the two different microbial communities were likely influenced by the different metabolic rate (based on ATP) measured in the two microbial communities. A higher metabolic activity can result in higher production of corrosive metabolites. In our study, as fermenters were the most abundant microbial groups it likely that higher metabolic activity resulted in higher concentration of acids in the test solution. An increased bacterial metabolic rate based has been previously associated as the main contributing factor in the acceleration of steel corrosion (Xu et al., 2022; Zhang et al., 2022). For example, Dzierzewicz et al. (1997), reported a statistically significant relationship between H_2_S release, bacterial growth rates, and, enzymatic activities rates (hydrogenases and ATPS-reductases) of *D. desulfuricans* with steel corrosion rates. It is worth mentioning that the synergy between higher concentrations of corrosive secondary metabolites being released by the biofilm developed in the untreated sand deposit, together with the physical barrier that the sand bed represents for the diffusion of these corrosive chemical species away from the metal surface, is very likely the main factor contributing to the high localized corrosion damaged observed in the metal surface under the untreated sand deposit.

Moreover, the higher corrosion rates found in the carbon steel exposed to untreated oilfield sand, together with the higher microbial diversity and activity found on it, suggests that MIC took place because of synergistic interactions among the different microbial species in the community. Higher relative abundances of H_2_ consuming microorganisms, such as sulfate reducers (*Desulfovibrio*) and thiosulfate reducers (*Oceanotoga*, *Shewanella*) were found in the untreated sand deposit, in comparison with the treated sand deposit. A syntrophic interaction among these microbial groups and fermenters have been previously studied (Laanbroek and Pfennig, 1981; Finke and Jørgensen, 2008). Fermenters secondary metabolites such as H_2_, can be used by sulfate and thiosulfate reducing bacteria as electron donors (Dar et al., 2008). Multispecies biofilms have been found more corrosive in comparison with single species biofilms due to the cascade of biochemical reactions that occur between taxonomically and metabolically different microorganisms (Zuo, 2007; Videla and Herrera, 2009).

Scanning electron microscope analysis demonstrated that despite the severity of the corrosion rates derived from the environment developed at the metal-deposit interface along the two sand deposits was different, the corrosion mechanisms revealed the grain boundaries in a low grade (micro-etching) in both tests, resembles the micro etching of carbon steel resulting from the standard practice of applying acid treatment previous to the microscopic examination of carbon steel (ASTM E407-07, 2007). Hence, results suggest that the UDMC mechanism is likely related with the organic acids released by bacteria as metabolic by-products, trapped in the metal-deposit interphase because of the tortuous pathways that the sand grains represent for their diffusion to the bulk solution. Most of bacterial acid metabolic byproducts are in the free acid form and are highly corrosive because their reduction coupled with the oxidation or iron is a thermodynamically favorable reaction and kinetically not retarded (Gu and Galicia, 2012).

The interaction of microorganisms with oilfield deposits is a topic that remains unexplored. In this paper, it is demonstrated that the chemical composition of one of the most commonly found deposits in oil and gas pipelines (sand), impacts the diversity, metabolic activity, and functional attributes of multispecies microbial communities, and consequently, affects the extent of under deposit microbial corrosion (UDMC). Results of this investigation provide valuable information about how microbial communities respond to different environmental conditions and how these microbiological changes impact the risk of corrosion. In addition, these results highlight the importance of including field samples in laboratory-based corrosion experiments to create systems that better simulate real field scenarios and therefore, generate more accurate corrosion risk assessments.

## 5. Conclusion

This investigation evaluated the effect of chemical differences of sand-deposits on the microbial community structure, functional attributes and metabolic activity of a multispecies oilfield microbial consortium, and its subsequent impact in under deposit-microbial corrosion. The main findings of this investigation are as follows:

1. Chemical characterization results of the raw untreated oilfield sand deposits and treated oilfield sand deposits showed that untreated sand contained organic compounds such as biocides, corrosion inhibitors, and petroleum hydrocarbons. In contrast, the roasted (treated) oilfield sand showed the absence of those chemical compounds. These chemical differences significantly affected diversity, richness, and evenness indexes. Untreated sand led to a more diverse, rich, and even microbial population, whereas a more homogenous, less rich, and uneven community resulted in the treated sand.
2. The taxonomic and functional attributes of the sessile microbial populations developed within the sand-deposit were also affected by the chemical differences between treated and untreated sand; (1) the microbial community in treated sand was dominated by fermenting species from only one genera, accompanied by low abundances of iron-oxidizers, thiosulfate reducers and sulfate reducers, moreover, the functional capability of the community evidenced a higher abundance of genes involved in carbohydrate and amino acid metabolism; (2) the microbial community in untreated sand was dominated by fermenting microorganisms of five (5) different genera, accompanied by moderate abundances of thiosulfate reducers, sulfate reducers and nitrate reducers, and, higher abundances of genes related to signal transduction and xenobiotics degradation were found on its functional capability prediction.
3. The presence of organic compounds such as biocides, petroleum hydrocarbons and corrosion inhibitors in the untreated sand significantly increased the average and localized corrosion induced by the multispecies oilfield consortium. Higher corrosion rates were correlated with higher ATP levels (microbial activity) in presence of these compounds, when compared with the treated sand. Higher corrosivity was attributed to the synergistic interactions that occurred between the diverse genera found in the untreated sand, which lead to a higher active microbial community, potentially producing higher concentrations and different corrosive metabolites, in comparison with the microbial community developed in the treated sand.
4. A correlation was found between the predicted microbial functional capability of the multispecies microbial consortium in the untreated sand (xenobiotics degradation), the chemical characterization of the untreated (xenobiotics presence), and higher corrosion rates in the UDMC test with untreated sand. The potential use of the organic compounds present in the sand as nutrients, by the multispecies microbial consortium and the associate risk of MIC requires further investigation.

## Data availability statement

The datasets presented in this study can be found in online repositories. The names of the repository/repositories and accession number(s) can be found at: https://www.ncbi.nlm.nih.gov/, PRJNA896746.

## Author contributions

MD-M, LM and SS-C contributed to conception and design of the study. MD-M executed the experiments and carried out the microbial, statistical, and corrosion analysis with support of SS-C. MD-M prepared the manuscript with the contribution of LM, SS-C, and HF. All authors contributed to the article and approved the submitted version.

## Funding

The authors declare that Qatar Environment and Energy Institute (QEERI) contributed financial resources to assist this work *via* a postgraduate scholarship. The study sponsor has reviewed and approved the submission of the manuscript for publication.

## Conflict of interest

The authors declare that the research was conducted in the absence of any commercial or financial relationships that could be construed as a potential conflict of interest.

## Publisher’s note

All claims expressed in this article are solely those of the authors and do not necessarily represent those of their affiliated organizations, or those of the publisher, the editors and the reviewers. Any product that may be evaluated in this article, or claim that may be made by its manufacturer, is not guaranteed or endorsed by the publisher.
