# Supplementary material for: Effect of deposit chemistry on microbial community structure and activity: Implications for under-deposit microbial corrosion
Source: Front Microbiol. 2023 Feb 9;14:1089649. doi: 10.3389/fmicb.2023.1089649 (PMC9947782; doi:10.3389/fmicb.2023.1089649)
Supplement: Supplementary file 1 [file Data_Sheet_1.docx]

Supplementary Material

# Supplementary Data


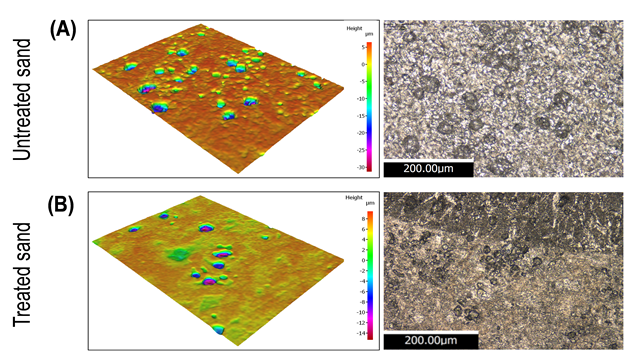


**Figure S1.** Visible light microscopy 3D images of carbon steel surface at 20X resolution evidencing localised deterioration of the metal. (A) coupons exposed to an untreated sand deposit, (B) coupons exposed to a treated sand deposit.

**Table S1**. p-value calculated from the one-way ANOVA test and Tukey’s post-hoc test for multiple comparisons of general corrosion rates among the treatments.

| Test | Comparison | Uniform corrosion rate   (p Value) |
| --- | --- | --- |
| Untreated | Treated | 0.0002 |

**Table S2**. p-value calculated from the one-way ANOVA test and Tukey’s post-hoc test for multiple comparisons of pitting rates among the treatments.

| Test | Comparison | Pitting rate  (p Value) |
| --- | --- | --- |
| Untreated | Treated | 0.0002 |

**Table S3**. p-value calculated from a t-test to determine the richness, diversity and evenness differences among the two sand deposits.

| Test | Comparison | Chao1 | Simpson | Shannon |
| --- | --- | --- | --- | --- |
| Treated | Untreated | 0.000 | 0.000 | 0.000 |

**Table S4**. Full list of LEfSe significant predicted pathways with sand-deposits type, LDA effect size and p values.

| **KEGG Pathway** | **Sand** | **Effect size** | **p-value** |
| --- | --- | --- | --- |
| Phenylalaninemetabolism | Treated | 2.679460118 | 0.049535 |
| Glucosinolatebiosynthesis | Treated | 2.304280289 | 0.049535 |
| Ribosome | Treated | 3.063846084 | 0.049535 |
| D_Alaninemetabolism | Treated | 2.43279238 | 0.049535 |
| Chloroalkaneandchloroalkenedegradation | Untreated | 2.471380852 | 0.049535 |
| Glycolysis_Gluconeogenesis | Treated | 3.091409792 | 0.049535 |
| Aminoacyl_tRNAbiosynthesis | Treated | 2.929734096 | 0.049535 |
| Glycerolipidmetabolism | Treated | 2.268251823 | 0.049535 |
| Pertussis | Untreated | 2.806863672 | 0.049535 |
| Ubiquinoneandotherterpenoid_quinonebiosynthesis | Untreated | 2.904895584 | 0.049535 |
| VitaminB6metabolism | Treated | 2.031311598 | 0.049535 |
| beta_Alaninemetabolism | Untreated | 2.682056213 | 0.049535 |
| Biosynthesisofaminoacids | Treated | 3.605102952 | 0.049535 |
| Carbonmetabolism | Treated | 3.548752219 | 0.049535 |
| Atrazinedegradation | Untreated | 2.246222667 | 0.049535 |
| Propanoatemetabolism | Treated | 2.228377339 | 0.049535 |
| Lipopolysaccharidebiosynthesis | Treated | 2.862388786 | 0.049535 |
| Glycine_serineandthreoninemetabolism | Treated | 3.239984465 | 0.049535 |
| Carbonfixationinphotosyntheticorganisms | Treated | 2.358325781 | 0.049535 |
| Citratecycle_TCAcycle_ | Treated | 2.804303758 | 0.049535 |
| Biosynthesisofenediyneantibiotics | Untreated | 2.078439063 | 0.049535 |
| Taurineandhypotaurinemetabolism | Treated | 2.51087266 | 0.049535 |
| Tryptophanmetabolism | Untreated | 2.535004731 | 0.049535 |
| Selenocompoundmetabolism | Treated | 2.191687346 | 0.049535 |
| Insecthormonebiosynthesis | Untreated | 2.165108444 | 0.049535 |
| Carbapenembiosynthesis | Untreated | 2.053866978 | 0.049535 |
| MetabolismofxenobioticsbycytochromeP450 | Untreated | 2.073200649 | 0.049535 |
| Benzoatedegradation | Untreated | 2.654334461 | 0.049535 |
| Valine_leucineandisoleucinedegradation | Treated | 2.249184548 | 0.049535 |
| Flavonoidbiosynthesis | Untreated | 2.315333758 | 0.036904 |
| Aminosugarandnucleotidesugarmetabolism | Treated | 2.346004729 | 0.049535 |
| Butanoatemetabolism | Treated | 2.195652278 | 0.049535 |
| Biosynthesisofantibiotics | Treated | 3.533310217 | 0.049535 |
| Flavoneandflavonolbiosynthesis | Untreated | 2.169664197 | 0.036904 |
| Biosynthesisofansamycins | Treated | 2.386417919 | 0.049535 |
| Homologousrecombination | Treated | 2.620162971 | 0.049535 |
| Lysinedegradation | Untreated | 2.523039974 | 0.049535 |
| Sulfurrelaysystem | Treated | 2.0043069 | 0.049535 |
| NOD_likereceptorsignalingpathway | Untreated | 2.537788145 | 0.049535 |
| Sphingolipidmetabolism | Untreated | 2.138744416 | 0.049535 |
| Biosynthesisofunsaturatedfattyacids | Untreated | 2.122720615 | 0.049535 |
| Quorumsensing | Treated | 3.643946956 | 0.049535 |
| Nitrogenmetabolism | Untreated | 2.355355032 | 0.049535 |
| Pyrimidinemetabolism | Treated | 2.7503526 | 0.049535 |
| Sulfurmetabolism | Untreated | 3.121050243 | 0.049535 |
| Otherglycandegradation | Untreated | 2.048720028 | 0.049535 |
| Proteasome | Treated | 2.203947525 | 0.049535 |
| Lysinebiosynthesis | Treated | 3.365414054 | 0.049535 |
| f_2_Oxocarboxylicacidmetabolism | Treated | 3.010536543 | 0.049535 |
| Antigenprocessingandpresentation | Untreated | 2.038335584 | 0.049535 |
| Phenylpropanoidbiosynthesis | Untreated | 2.133807997 | 0.049535 |
| Fattyaciddegradation | Untreated | 2.865048555 | 0.049535 |
| Stilbenoid_diarylheptanoidandgingerolbiosynthesis | Untreated | 2.386856888 | 0.036904 |
| Carbonfixationpathwaysinprokaryotes | Treated | 2.100599896 | 0.049535 |
| Fattyacidmetabolism | Untreated | 2.84910092 | 0.049535 |
| C5_Brancheddibasicacidmetabolism | Treated | 2.424730307 | 0.049535 |
| Biosynthesisofvancomycingroupantibiotics | Treated | 2.210931251 | 0.049535 |
| Glycosaminoglycandegradation | Untreated | 2.080560226 | 0.049535 |
| Pyruvatemetabolism | Treated | 2.809190313 | 0.049535 |
| Thermogenesis | Untreated | 2.346536599 | 0.049535 |
| D_ArginineandD_ornithinemetabolism | Treated | 2.172030433 | 0.049535 |
| Glyoxylateanddicarboxylatemetabolism | Treated | 3.074734558 | 0.049535 |
| Photosynthesis | Untreated | 2.648757704 | 0.049535 |
| Huntingtonsdisease | Untreated | 2.218396328 | 0.049535 |
| Pathwaysincancer | Untreated | 2.369807463 | 0.049535 |
| RNAtransport | Treated | 2.365289174 | 0.049535 |
| Microbialmetabolismindiverseenvironments | Treated | 3.53321548 | 0.049535 |
| RNAdegradation | Untreated | 2.225715047 | 0.049535 |
| Lipoicacidmetabolism | Treated | 2.329520488 | 0.049535 |
| Aldosteronesynthesisandsecretion | Untreated | 2.043543495 | 0.049535 |
| Flagellarassembly | Untreated | 2.04073552 | 0.049535 |
| Cationicantimicrobialpeptide_CAMP_resistance | Untreated | 2.977496126 | 0.049535 |
| Plant_pathogeninteraction | Untreated | 2.05711982 | 0.049535 |
| Cyanoaminoacidmetabolism | Treated | 2.233567608 | 0.049535 |
| Caprolactamdegradation | Untreated | 2.261270455 | 0.049535 |
| Purinemetabolism | Treated | 3.34418686 | 0.049535 |
| Peroxisome | Untreated | 2.013037378 | 0.049535 |
| Carbohydratedigestionandabsorption | Untreated | 2.096542433 | 0.049535 |
| Bacterialchemotaxis | Untreated | 3.338235152 | 0.049535 |
| HepatitisB | Untreated | 2.408805226 | 0.049535 |
| Longevityregulatingpathway_multiplespecies | Untreated | 2.381748162 | 0.049535 |
| Cellcycle_Caulobacter | Untreated | 3.300549529 | 0.049535 |
| Peptidoglycanbiosynthesis | Treated | 2.94881179 | 0.049535 |
| Argininebiosynthesis | Treated | 2.827737743 | 0.049535 |
| Metabolicpathways | Treated | 3.92483158 | 0.049535 |
| Proteinexport | Treated | 2.173150002 | 0.049535 |
| Biofilmformation_Escherichiacoli | Untreated | 3.380921667 | 0.049535 |
| Penicillinandcephalosporinbiosynthesis | Untreated | 2.143602299 | 0.049535 |
| EpithelialcellsignalinginHelicobacterpyloriinfection | Untreated | 2.261730767 | 0.049535 |
| Tropane_piperidineandpyridinealkaloidbiosynthesis | Treated | 2.122175133 | 0.049535 |
| Degradationofaromaticcompounds | Untreated | 2.540026393 | 0.049535 |
| Bacterialsecretionsystem | Untreated | 2.08944776 | 0.049535 |
| RNApolymerase | Treated | 2.151756964 | 0.049535 |
| beta_Lactamresistance | Treated | 2.363819428 | 0.049535 |
| Oxidativephosphorylation | Treated | 2.986486386 | 0.049535 |
| Glycosphingolipidbiosynthesis_ganglioseries | Untreated | 2.027554907 | 0.036904 |
| Ascorbateandaldaratemetabolism | Untreated | 2.339625626 | 0.049535 |
| Fructoseandmannosemetabolism | Treated | 2.710079089 | 0.049535 |
| Riboflavinmetabolism | Untreated | 2.148721674 | 0.049535 |
| Fattyacidbiosynthesis | Untreated | 2.452354526 | 0.049535 |
| Biofilmformation_Vibriocholerae | Untreated | 3.514892827 | 0.049535 |
| Glucagonsignalingpathway | Treated | 2.003336102 | 0.049535 |
| Polyketidesugarunitbiosynthesis | Treated | 2.202787708 | 0.049535 |
| Novobiocinbiosynthesis | Treated | 2.292147114 | 0.049535 |
| Phenylalanine_tyrosineandtryptophanbiosynthesis | Treated | 2.230635934 | 0.049535 |
| Histidinemetabolism | Treated | 2.908806536 | 0.049535 |
| Pentosephosphatepathway | Treated | 2.613541189 | 0.049535 |
| Cushingssyndrome | Untreated | 2.022625385 | 0.049535 |
| HIF_1signalingpathway | Treated | 2.037978764 | 0.049535 |
| Fluidshearstressandatherosclerosis | Untreated | 2.29837921 | 0.049535 |
| Cysteineandmethioninemetabolism | Treated | 2.884870296 | 0.049535 |
| Two_componentsystem | Untreated | 4.359496425 | 0.049535 |
| Limoneneandpinenedegradation | Untreated | 2.429532432 | 0.049535 |
| Porphyrinandchlorophyllmetabolism | Untreated | 3.109191666 | 0.049535 |
| Valine_leucineandisoleucinebiosynthesis | Treated | 2.818192769 | 0.049535 |
| PantothenateandCoAbiosynthesis | Treated | 2.713198496 | 0.049535 |
| Phosphotransferasesystem_PTS_ | Treated | 3.064134948 | 0.049535 |
| Biosynthesisofsecondarymetabolites | Treated | 3.45309161 | 0.049535 |
| Phosphonateandphosphinatemetabolism | Untreated | 2.472091238 | 0.049535 |
| Apoptosis_fly | Untreated | 2.302578748 | 0.049535 |
| Galactosemetabolism | Untreated | 2.087015076 | 0.049535 |
| Pentoseandglucuronateinterconversions | Treated | 2.970475857 | 0.049535 |
| Mismatchrepair | Untreated | 2.237644686 | 0.049535 |
| Biosynthesisofsiderophoregroupnonribosomalpeptides | Untreated | 2.324970252 | 0.049535 |
| Cardiacmusclecontraction | Untreated | 2.010230524 | 0.049535 |
| Vancomycinresistance | Treated | 2.532683126 | 0.049535 |
| Nicotinateandnicotinamidemetabolism | Treated | 2.528703223 | 0.049535 |
| Folatebiosynthesis | Treated | 2.406336533 | 0.049535 |
| Geranioldegradation | Untreated | 2.151682606 | 0.049535 |
| Nucleotideexcisionrepair | Treated | 2.59278286 | 0.049535 |
| Alzheimersdisease | Untreated | 2.056014769 | 0.049535 |
| Methanemetabolism | Treated | 2.833358887 | 0.049535 |
| Biofilmformation_Pseudomonasaeruginosa | Untreated | 3.663508693 | 0.049535 |
| Drugmetabolism_cytochromeP450 | Untreated | 2.00085854 | 0.049535 |
| Onecarbonpoolbyfolate | Treated | 2.529484311 | 0.049535 |
| Salmonellainfection | Untreated | 2.999378378 | 0.049535 |

**Table S5.** p-value calculated from the one-way ANOVA test and Tukey’s post-hoc test for multiple comparisons of ATP concentration among the biotic treatments.

| Test | Comparison | ATP (p Value) |
| --- | --- | --- |
| Treated | Untreated | 0.0000 |
